# A Comprehensive Evaluation of Zirconia-Reinforced Glass Ionomer Cement’s Effectiveness in Dental Caries: A Systematic Review and Network Meta-Analysis

**DOI:** 10.3390/dj11090211

**Published:** 2023-09-08

**Authors:** Srikurmam Manisha, Soumya S Shetty, Vini Mehta, Rizwan SA, Aida Meto

**Affiliations:** 1 Department of Conservative Dentistry & Endodontics, Dr. D. Y. Patil Dental College and Hospital, Dr. D. Y. Patil Vidyapeeth, Pimpri, Pune 411018, Maharashtra, India; soumya.shetty@dpu.edu.in (S.S.S.); aidameto@yahoo.com (A.M.); 2 Department of Dental Research Cell, Dr. D. Y. Patil Dental College and Hospital, Dr. D. Y. Patil Vidyapeeth, Pimpri, Pune 411018, Maharashtra, India; 3 Scientist-D, ICMR-National Institute of Epidemiology, Chennai 600077, India; sarizwan1986@gmail.com; 4 Department of Dentistry, Faculty of Dental Sciences, University of Aldent, 1007 Tirana, Albania; 5 Clinical Microbiology, School of Dentistry, University of Modena and Reggio Emilia, 41125 Modena, Italy

**Keywords:** dental caries, novel glass ionomer cement, tooth restoration, zirconia, in vitro

## Abstract

Dental cements are in a constant state of evolution, adapting to better align with the intricacies of tooth structure and the dynamic movements within the oral cavity. This study aims to evaluate the efficacy of zirconia-reinforced glass ionomer cement—an innovative variant of modified glass ionomer cements—in terms of its ability to withstand compressive forces and prevent microleakage during dental caries reconstruction. An extensive search was conducted across various databases, encompassing PubMed-MEDLINE, Scopus, Embase, Google Scholar, prominent journals, unpublished studies, conference proceedings, and cross-referenced sources. The selected studies underwent meticulous scrutiny according to predetermined criteria, followed by the assessment of quality and the determination of evidence levels. In total, 16 studies were incorporated into this systematic review and network meta-analysis (NMA). The findings suggest that both compomer and giomer cements exhibit greater compressive strength and reduced microleakage values than zirconia-reinforced glass ionomer cement. In contrast, resin-modified glass ionomer cement (RMGIC) and high-viscosity glass ionomer cement (GIC) demonstrate less favorable performance in these regards when compared with zirconia-reinforced glass ionomer cement.

## 1. Introduction

Dental caries involves the localized destruction of tooth substance, encompassing both organic and inorganic components, leading to the emergence of various observable symptoms. This condition results from the accumulation of plaque, triggering alterations in the microbial environment and subsequently causing localized shifts in pH [1]. Consequently, timely identification and treatment are advised to prevent possible complications if disease progression reaches the pulp chamber. The initial stages of dental caries typically manifest as white spot lesions, which can progress to discoloration without timely intervention [2]. Therapeutic restorative treatment is aimed at eliminating the carious lesion and reinstating the tooth’s original contours and contacts [3]. With the introduction of adhesive restorative materials, clinicians have the opportunity to choose conservative preparations that facilitate the preservation of maximum tooth structure, while also ensuring aesthetic concern, given that the majority of adhesive restorations are designed to match the natural tooth color [3,4]. Dental cements represent restorative materials composed of small biomolecules that demonstrate biocompatibility and contribute positively to tooth structure. These materials ideally possess qualities such as tooth adhesion, antimicrobial attributes, and resilience against masticatory forces during normal function and parafunction [5]. Glass ionomer cement (GIC), also known as “polyalkenoate cement”, is among the earliest dental cements with the unique attributes of fluoride release and adherence to tooth structure [6,7]. These cements are consistently fortified through the incorporation of fine particles, enhancing their ability to withstand higher forces without compromising aesthetics [8,9].

Despite extensive clinical experience, these cements still exhibit certain limitations [10]. Compressive strength stands as a pivotal factor to be considered within the oral cavity’s dynamic environment. This strength signifies a restorative material’s resistance to intraoral forces, including compressive and tensile stresses generated by functional and parafunctional movements [11]. Testing often serves to predict a restorative material’s clinical longevity [12]. Conventional GICs typically demonstrate compressive strengths ranging from 150 to 220 MPa [10,13]. While achieving properties akin to natural teeth is impractical, efforts should be directed toward refining properties to enhance clinical durability [14]. During the setting process, adhesive materials may undergo either shrinkage or expansion. Materials that undergo shrinkage encounter difficulties in effectively sealing against the tooth surface, potentially leading to the infiltration of bacteria [3]. This phenomenon, known as microleakage, is frequently manifested as marginal staining, postoperative sensitivity, and the development of secondary caries around the restoration site [15]. Consequently, the selection of a restorative material should prioritize its adaptability to the tooth structure.

The main objective of this study was to conduct a thorough evaluation of the effectiveness of zirconia-reinforced glass ionomer cement, an innovative variant of modified glass ionomer cements, in the context of dental caries reconstruction. Our specific aims included a detailed assessment of its mechanical properties, notably compressive strength, and its ability to prevent microleakage. We pursued this goal through a comprehensive systematic review and network meta-analysis, directly comparing zirconia-reinforced glass ionomer cement with conventional modified glass ionomer cements. Our ultimate aim was to contribute evidence-based insights that can inform clinical decision-making regarding the efficacy of this material in restoring teeth impacted by carious lesions.

## 2. Materials and Methods

The protocol for this current review was registered and assigned the identification number CRD42022310393 in the PROSPERO database, maintained by the National Institute for Health Research, University of York, Center for Reviews and Dissemination. The preparation of this manuscript adheres to the Preferred Reporting Items for Systematic Reviews and Meta-Analysis Extension (PRISMA) statement, designed for systematic reviews that incorporate network meta-analyses [16,17]. It adheres to evidence based practice for clinical question [18].

### 2.1. Inclusion Criteria

In vitro studies conducted on extracted human teeth within the time frame of 1 January 2015 to 1 January 2022 were included. The studies employed cement specimens and focused on the comparison of compressive strength and microleakage properties between zirconia-reinforced glass ionomer cement and modified glass ionomer cements.

### 2.2. Exclusion Criteria 

Retrospective clinical studies, case reports, animal studies, and studies that did not measure compressive strength and microleakage properties were excluded. 

### 2.3. Search Methods and Data Collection 

A comprehensive literature search encompassing major electronic databases—PubMed-MEDLINE, Scopus, and Embase—was supplemented with additional sources, including Google Scholar, notable journals, unpublished studies, conference proceedings, and cross-references. The search, conducted from 1 January 2015, to 1 January 2022, employed an exhaustive query (Table S1) to identify eligible studies, utilizing keywords such as “cement specimen”, “zirconia-reinforced glass ionomer cement”, “modified glass ionomer cement”, “compressive strength”, and “microleakage”. Initially developed for the MEDLINE database, the search strategy was constructed through a blend of medical subject headings (MeSH) and accessible text terms, later adapted as necessary for other databases. Results were imported into a bibliographic database to facilitate deduplication, while cross-references were meticulously reviewed. Consistency was maintained in applying a publication date filter across all searches. Two authors independently executed the literature search, assessed the study articles, and extracted pertinent data. This review process consisted of two stages. Firstly, the titles and abstracts of all articles were scrutinized, followed by a meticulous evaluation of full-text content. Studies aligning with the selection criteria underwent subsequent data extraction. Employing tailored data extraction forms within Microsoft Excel, the two authors independently collected relevant information. Any differences in extraction were resolved through constructive dialogue between the authors. For each chosen study, the subsequent information was extracted from a standardized form, where available: author and year of publication, study design, tested properties, participant demographics, interventions, comparators, intervention group sample size, comparator group sample size, specimen dimensions, testing methodologies, dye utilization, microscopic analysis, scoring criteria, and author’s conclusions.

### 2.4. Quality Assessment and Risk of Bias

The Joanna Briggs Institute (JBI) Critical Appraisal tool [19] and the Checklist for Reporting In Vitro Studies (CRIS) [20] were utilized to evaluate the risk of bias in eligible studies. These tools were customized, involving the addition or removal of specific questions to align with the reviewers’ judgment. The assessment encompassed various parameters crucial for evaluating study quality. These included: detailing of sample size calculations, randomization of teeth, comparable baseline properties across treatment groups, preparation of samples by a single operator, operator blinding, uniform measurement of outcome groups, assessor blinding, consistent outcome measurement, appropriate statistical analysis, comprehensive result reporting for all samples, and the absence of other biases in the study design. For each parameter, its presence was noted as “YES” and absence as “NO”. Then, the classification of studies based on risk of bias was determined as follows: studies with 1–5 “YES” responses were categorized as having a high risk of bias, those with 6–7 “YES” responses as having a moderate risk, and those with 8 or more “YES” responses as having a low risk. This assessment was independently conducted by two authors. Resolutions were achieved through collective deliberation among all authors to ensure unanimous decisions. Notably, no studies were excluded on the grounds of risk of bias.

### 2.5. Quantitative Analysis and Synthesis of the Network Meta-Analysis (NMA)

The quantitative evaluation aimed to assess and juxtapose zirconia-reinforced glass ionomer cements and modified glass ionomer cements in relation to their compressive strength and microleakage attributes for dental caries reconstruction. For each outcome, we computed the mean difference (MD) and established the corresponding 95% confidence interval (CI) for both compressive strength and microleakage. The effectiveness of these prespecified outcomes was determined through the comparison of zirconia with other compounds. By leveraging the Netmeta package within the R programming environment, a random-effects network meta-analysis was conducted individually for the two outcomes. Heterogeneity was characterized using the I2 statistic. To showcase the interrelations among various studies, network plots were provided, along with league tables presenting the MD and 95% CI for every conceivable compound comparison. Illustrative forest plots were employed to graphically portray the mean difference between compounds, while funnel plots demonstrated the potential extent of publication bias. A ranking of treatments from highest to lowest was facilitated using *p*-score statistics. To assess the proportion of direct and indirect evidence, evidence plots (Figure S1) were generated for each comparison, accompanied by the calculation of mean path length and parallelism. Additionally, split plots were crafted to compare direct, indirect, and network-level estimates for each comparison (Figure S2). To gauge the consistency within the network, heat plots were constructed for possible comparisons (Figure S3). A *p*-value of <0.05 was considered statistically significant, and all analyses were executed using R software version 4.1.2.

## 3. Results

### 3.1. Search Results and Study Selection

**Figure 1:**
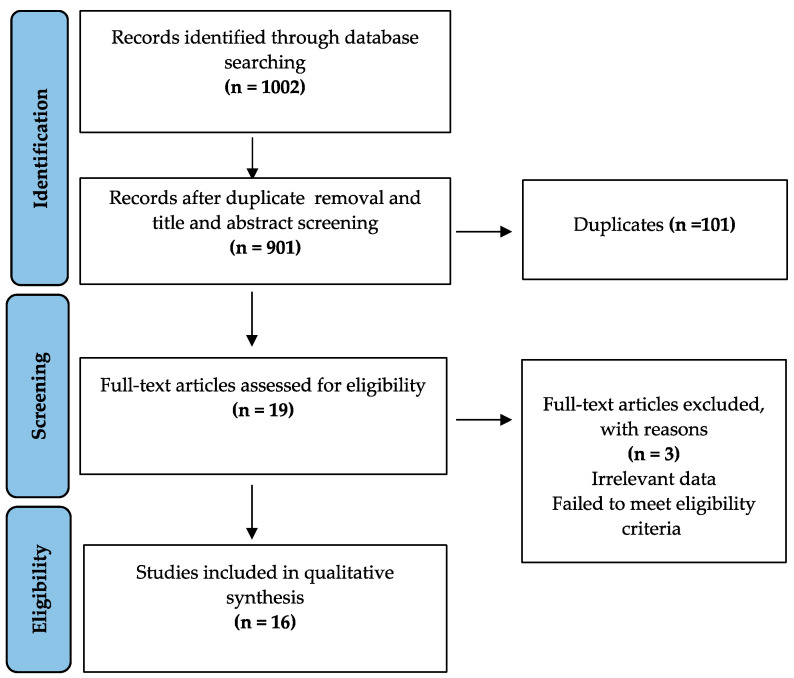
PRISMA flowchart summarizing the process of article selection (n, number of studies).

The process of literature search is depicted in Figure 1 (PRISMA flowchart). Initially, a total of 1002 pertinent articles were pinpointed via electronic databases and manual exploration. Upon eliminating duplicates, 901 articles underwent title and abstract screening. Following this stage, a thorough evaluation of 19 full-text articles was undertaken, resulting in the inclusion of 16 studies [21,22,23,24,25,26,27,28,29,30,31,32,33,34,35,36], which encompassed systematic reviews and network meta-analyses.

### 3.2. Study Characteristics

Among the 16 studies incorporated in the review, 7 [22,23,24,25,26,27,28] solely assessed compressive strength, while 1 study [21] evaluated both compressive strength and microleakage. Within this subset, eight studies [29,30,31,32,33,34,35,36] concentrated solely on microleakage. The intervention across all studies encompassed zirconia-reinforced glass ionomer cements, including variations like zirconomer and zirconomer-improved glass ionomer cements, available commercially. As comparators, modified glass ionomer cements were employed, specifically high-viscosity GICs (commercially known as ketac molar, type IX, and type IX extra GICs), giomer (commercially recognized as beautifil II cement), silver-reinforced glass ionomer cement (commercially referred to as Xtracem-S, miracle mix), compomer (commercially identified as dyract-XP, compoglass F), glass hybrid (commercially designated as equia forte), amalgomer CR, resin-modified glass ionomer cement (commercially available as Fuji II LC capsule), nano-ionomer, and glass carbomer (commercially labeled as glassfill). For a detailed composition of each cement considered within this review, please refer to Table S2.

#### 3.2.1. Description of Compressive Strength Studies

The studies conducted during the period of 2016 to 2020 employed a comparative cross-sectional study design. The majority of these studies featured a sample size of 10. Notably, all studies employed cylindrical cement specimens, albeit with varying measurements. The number of comparators ranged from one in two studies [24,25] to two in three studies [21,22,27], three in one study [26], four in another [28], and five in a single study [23]. In the context of specific cement types, five studies encompassed high-viscosity glass ionomer cements [21,22,24,25,26,28], while four studies involved giomer [21,23,26,28]. For silver-reinforced glass ionomer cement, there was one study [22], and similarly, one study pertained to glass carbomer [28]. Resin-modified glass ionomer cement [23,27], compomer [23,26], glass hybrid [23,27], and amalgomer CR [23,28] each featured in two studies. Across these investigations, the findings generally suggested that the compressive strength of giomer, compomer, and high-viscosity glass ionomer cements surpassed that of zirconia-reinforced glass ionomer cement. An overview of studies examining compressive strength is provided in Table 1.

#### 3.2.2. Description of Microleakage Studies

All studies incorporated within this analysis were conducted between 2017 and 2020, adopting an in vitro study design. The majority of these studies featured a sample size of 10. Notably, the number of comparatives in these studies exhibited variability: one comparative in five studies [21,29,30,33,35], two comparatives in three studies [33,34,36], and three comparatives in one study [31]. Turning to specific cement types, three studies encompassed high-viscosity glass ionomer cement [21,29,36], three studies centered around giomer [31,35], and three studies were dedicated to resin-modified glass ionomer cement [31,32,33]. For silver-reinforced glass ionomer cement [30,36] and glass hybrid [32,34], two studies were available. A single study each pertained to glass carbomer [34] and nano-ionomer [31]. Diverse test types were employed across these studies, ranging from dye penetration in eight studies [21,30,31,32,33,34,35,36] to dye absorbance in one study [29]. The dyes utilized encompassed methylene blue, silver paint, silver nitrate, and basic fuchsine. The methodologies of microscopy varied, with spectrophotometry in one study [29], stereomicroscopy in seven studies [21,30,31,32,33,34,35], and scanning electron microscopy in another [36]. Distinct criteria were employed for microscopy evaluation in each study. It is worth noting that the findings of a significant number of studies suggested that the values associated with zirconia-reinforced glass ionomer cement exceeded those of the other cement types. A comprehensive summary of studies addressing microleakage can be found in Table 2.

### 3.3. NMA Synthesis

**Figure 2:**
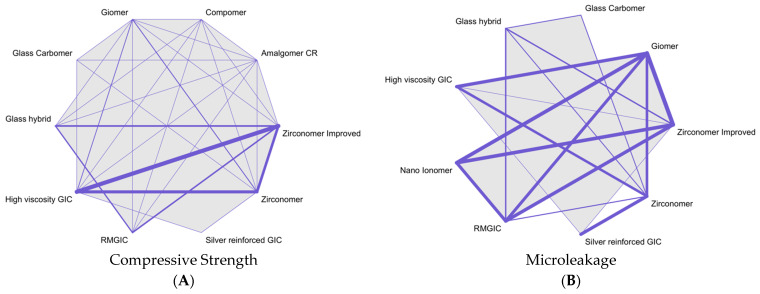
Network meta-analysis of eligible comparisons for (**A**) compressive strength and (**B**) microleakage. The thickness of lines between the interventions relates to the number of studies for that comparison. GIC: glass ionomer cement; RMGIC: resin-modified glass ionomer cement; Amalgomer CR: ceramic-reinforced glass ionomer cement.

The network meta-analysis encompassed the evaluation of both compressive strength (measured in megapascals) and microleakage. The mean intervention and comparative values for compressive strength and microleakage are presented in Table S3. Throughout all networks, the guiding principles of coherency, transitivity, and consistency were maintained. Figure 2 provides visual insight into the NMA maps detailing the investigation of the efficacy of zirconia-reinforced glass ionomer cements in comparison with that of modified glass ionomer cements. The thickness of the lines connecting interventions reflects the number of studies within each connection.

More in-depth information regarding the influence of compressive strength and microleakage on each NMA, alongside direct and indirect comparisons, is available in Table S3 and Figure S1. Comprehensive matrices of results are provided in Table 3 and Table 4, while Table S4 ranks various materials based on compressive strength and microleakage.

#### 3.3.1. Compressive Strength

**Figure 3:**
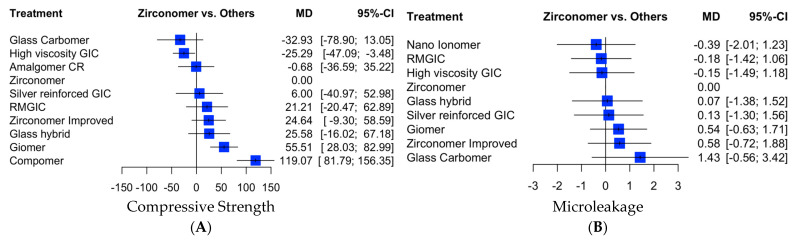
Forest plots for (**A**) compressive strength (**B**) microleakage. GIC: glass ionomer cement; RMGIC: resin-modified glass ionomer cement; Amalgomer CR: ceramic-reinforced glass ionomer cement.

In the forest plots presented here, values situated to the left of the vertical line “0” signify lower values than those of zirconia-reinforced glass ionomer cement, while values associated with cements positioned to the right of the line (compomer, giomer) are higher than those of zirconia-reinforced glass ionomer cement, which is a desirable outcome. The values attributed to cements such as amalgomer CR, zirconomer, zirconomer-improved, silver-reinforced GIC, and glass hybrid cements align at a comparable level (Figure 3A).

**Figure 4:**
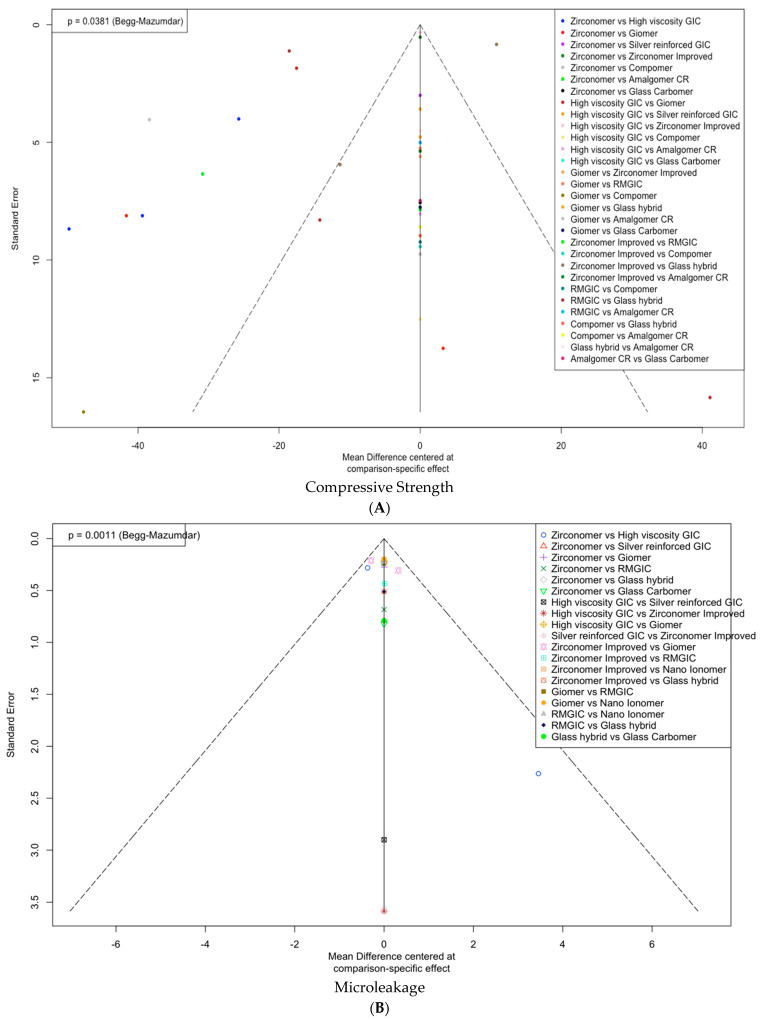
Funnel plots for (**A**) compressive strength and (**B**) microleakage. GIC: glass ionomer cement; RMGIC: resin-modified glass ionomer cement; Amalgomer CR: ceramic-reinforced glass ionomer cement.

Funnel plots were employed to assess the presence of publication bias in studies comparing the compressive strength of zirconia-reinforced glass ionomer cements (zirconomer, zirconomer-improved) with that of modified glass ionomer cements (high-viscosity glass ionomer cements—ketac molar, type IX, type IX extra, giomer, compomer; silver-reinforced glass ionomer cements—miracle mix, Xtracem-S, resin-modified glass ionomer cement; glass hybrid—equia forte; glass carbomer—glassfill, amalgomer CR, nano-ionomer). The calculated *p*-value for the Begg–Mazumdar test was 0.0381, indicating evidence of potential publication bias (Figure 4A). In addition, Figure S2 illustrates both direct and indirect comparisons of compressive strength.

#### 3.3.2. Microleakage

In forest plot presented here, cement values to the left of the vertical line at “0” (nano-ionomer, RMGIC, high-viscosity GIC) are lower, which is desirable. Conversely, the values of cements positioned to the right of the vertical line (glass hybrid, silver-reinforced GIC, giomer, zirconomer-improved, and glass carbomer) are higher and considered undesirable (Figure 3B). Figure S2B illustrates direct and indirect comparisons for microleakage. A funnel plot highlights the presence of potential publication bias in studies comparing the microleakage of zirconia-reinforced glass ionomer cements (zirconomer, zirconomer-improved) with that of modified glass ionomer cements (high-viscosity glass ionomer cements—ketac molar, type IX, type IX extra, giomer, compomer; silver-reinforced glass ionomer cements—miracle mix, Xtracem-S, resin-modified glass ionomer cement; glass hybrid—equia forte; glass carbomer—glassfill, Amalgomer CR, nano-ionomer). The calculated *p*-value for the Begg–Mazumdar test was 0.0011 (Figure 4B). In Figure S3A, for compressive strength, the field colors range from deep red (indicating substantial inconsistency) to blue (indicating that evidence from this design supports evidence in the row). Figure S3B indicates that grey boxes signify the importance of a treatment comparison for estimating another treatment comparison for microleakage. A common finding is that boxes are prominent in the heatmap’s diagonal axis, implying the utilization of direct evidence. The colored backgrounds indicate inconsistent design in a row, attributed to the design in a column.

### 3.4. Assessment of Risk of Bias

A comprehensive risk of bias assessment was carried out for all the studies included in the analysis. Out of these, thirteen studies [21,22,23,25,26,27,28,30,32,33,34,35,36] were found to have a moderate risk of bias, while two studies [24,31] were identified as having a high risk of bias. In contrast, only one study [29] showed a low risk of bias. None of the studies reported sample size calculations or the blinding of operators. Conversely, all the studies indicated similarity of treatment groups at baseline, standardization of procedures, outcome measurement, appropriate statistical analysis usage, and reporting of results for all samples. Furthermore, three studies [29,30,34] mentioned that the same operator treated all the samples, and only two studies [29,32] clarified the blinding of outcome assessors. Table S5 provides a summary of the quality assessment for individual studies.

## 4. Discussion

In the following, we will discuss our main findings revealed by this extensive meta-analysis. The central aim is to investigate the characteristics of various glass ionomer cements, specifically focusing on their clinically significant aspects such as compressive strength and microleakage. Through this analysis, we gain valuable insights into the nuanced properties of these cements, challenging the notion that newer variants invariably outperform their predecessors. Advancements in adhesive restorative materials have enabled smaller cavity preparations to preserve more tooth structure [37]. Among the assortment of cements scrutinized, two varieties, compomer and giomer, emerge as standout contenders boasting superior strength characteristics [38,39]. This strength superiority can be attributed to inherent factors—giomer’s pre-reacted components and compomer’s resin constituents—both contributing to their robustness in resisting mechanical forces. Additionally, the presence of a resin element enhances the cement’s early setting, leading to reduced instances of microleakage compared to other counterparts. Before we proceed to conduct a thorough comparison of these attributes, it is crucial to develop a comprehensive understanding of the evolutionary trajectory of the composition of each type of cement. Exploring the distinct compositions that underlie these various cements will enable readers to better comprehend the comparative attributes and draw more meaningful conclusions.

Glass ionomer cements continue to evolve, addressing previous challenges. One approach involves incorporating silver alloy particles into ionomer glass portions and fusing silver powder particles with glass to create a metal-modified glass ionomer cement. Other variations, such as “hybrid ionomers” and “resin-modified glass ionomer cements”, use resin and monomer systems, enhancing aesthetics and clinical performance [2,40,41]. New dental cements have emerged, including highly viscous glass ionomers with increased glass filler particles for higher strength [42,43]. Compomers, combining glass ionomers and resin composites, demonstrate improved mechanical properties but still lag behind resin-based composites [44,45]. Giomers, resin ionomers with pre-reacted glass fillers, offer strength and fluoride release [46]. However, properties of glass carbomer, glass hybrid, and amalgomer CR remain inferior to those of recently introduced GICs and resin-based GICs [47].

Zirconomer and zirconomer-improved cements, as well as zirconia-reinforced glass ionomers, possess the mechanical strength of amalgam restorations and fluoride leaching properties [48]. Improved mechanical properties are attributed to added zirconium fillers [49,50]. Controlled micro-ionization results in uniform particle sizes, enhancing final strength and clinical durability [12,43,48,51]. Predicting clinical longevity requires testing compressive strength. Anterior teeth require pleasing aesthetics, while posterior restorations demand mechanical resilience [52,53,54]. Lower-strength cements may lead to restoration failure [55,56,57,58]. Prabhakar et al. found silver-reinforced glass ionomer cement’s compression strength superior to that of conventional glass ionomer due to silver particles promoting gelation [59]. The results of this study are per many other studies [60,61,62,63,64,65,66]. Chalissery et al. and Dheeraj et al. offer differing conclusions. Zirconomer’s higher compressive strength and adequate fluoride release make it valuable for small to medium cavities and high-risk patients [28,67,68].Reduced microleakage values are crucial to prevent saliva and microorganism entry [69,70,71]. If the restoration fails to adapt closely to the tooth structure, it allows entry of saliva and microorganisms, which play a role in secondary disease initiation and progression [69,71,72]. Hence, lower microleakage values are needed in a restorative material [73,74,75,76], and marginal microleakage should be evaluated for any restorative material as it directly translates to the success or failure of the restorations [73,74,75,76]. Microleakage testing methods include dye penetration and various techniques [77,78,79,80,81,82,83]. Baghdadi et al. [84] and Albeshti et al. [12] emphasize microleakage’s impact.

Higher microleakage values in zirconomer might result from zirconia filler particles affecting chelation reactions [12,85]. Despite this, no restorative material entirely prevents microleakage [29,30,31,32,33,34,35,36]. Understanding clinical behavior based on in vitro and in vivo studies is crucial for successful restorations [12,21,22,23,24,25,26,27,28,29,30,31,32,33,34,35,36,86,87]. Factors such as compressive strength and microleakage are pivotal in material selection. This systematic review and meta-analysis aim to explore contemporary material properties, particularly those of zirconomer. However, further in vivo studies and clinical trials are essential to solidify findings from in vitro studies.

Study limitations include unclear effects of different brands and sample sizes on compressive strength and microleakage values. While cement classes are likely have similar compositions, minor filler particle variations exist. Microleakage outcomes vary due to different dyes and microscopy methods, introducing potential heterogeneity.

## 5. Conclusions

From our network meta-analysis results, we have ascertained that zirconia-reinforced glass ionomer cement has lower compressive strength than compomer and giomer cements. However, there are no significant differences when compared with other modified cements. The microleakage values of zirconia-reinforced glass ionomer cements are similar to those of other cements, with no notable distinctions. Consequently, the properties of zirconia-reinforced glass ionomer cements are on par with those of the comparative compounds in our review. This type of cement holds potential as a practical substitute for modified glass ionomer cements, owing to its pleasing aesthetics and significant clinical benefits. Clinicians should familiarize themselves with available restorative materials and understand their pros and cons. It iss essential to note that further in vivo studies are necessary to confirm the initial observations from in vitro studies.

## Figures and Tables

**Table 1:** Characteristics of the studies included regarding compressive strength.

Author/Year	Study Design	Property Tested	Population	Intervention	Comparative	Sample Size	Dimensions of Specimen
Walia R/2016 [21]	Comparative cross-sectional study	Compressive strength	Cylindrical cement specimens	Zirconomer	High-viscosity GIC	15	5 mm × 6 mm
					Giomer		5 mm × 6 mm
Bhatia H/2017 [22]	Comparative cross-sectional study	Compressive strength	Cylindrical cement specimens	Zirconomer	High-viscosity GIC	15	4 mm × 6 mm
					Silver-reinforced GIC		4 mm × 6 mm
Canturk K/2020 [23]	Comparative cross-sectional study	Compressive strength	Cylindrical cement specimens	Zirconomer-improved	RMGIC	10	4 mm × 6 mm
					Compomer		4 mm × 6 mm
					Glass hybrid		4 mm × 6 mm
					Giomer		4 mm × 6 mm
					Amalgomer CR		4 mm × 6 mm
Shetty C/2017 [24]	Comparative cross-sectional study	Compressive strength	Cylindrical cement specimens	Zirconomer	High-viscosity GIC	10	3 mm × 6 mm
				Zirconomer-improved			3 mm × 6 mm
Patel A/2018 [25]	Comparative cross-sectional study	Compressive strength	Cylindrical cement specimens	Zirconomer	High-viscosity GIC	10	6 mm × 12 mm
Patil K/2016 [26]	Comparative cross-sectional study	Compressive strength	Cylindrical cement specimens	Zirconomer	Giomer	5	5 mm × 5 mm
					High-viscosity GIC		5 mm × 5 mm
					Compomer		5 mm × 5 mm
S Dhivya/2017-2020 [27]	Comparative cross-sectional study	Compressive strength	Cylindrical cement specimens	Zirconomer-improved	Glass Hybrid	15	4 mm × 6 mm
					RMGIC		4 mm × 6 mm
Uğurlu M/2020 [28]	Comparative cross-sectional study	Compressive strength	Cylindrical cement specimens	Zirconomer	Glass Carbomer	10	4 mm × 6 mm
					Amalgomer CR		4 mm × 6 mm
					High-viscosity GIC		4 mm × 6 mm
					Giomer		4 mm × 6 mm

GIC: glass ionomer cement; RMGIC: resin-modified glass ionomer cement; Amalgomer CR: ceramic-reinforced glass ionomer cement.

**Table 2:** Characteristics of the studies included in microleakage analysis.

Author/Year	Study Design	Property Tested	Population	Intervention	Comparative	Sample Size	Test Type Used	Dye Used	Microscopy
Asafarlal S/2017 [29]	In vitro study	Microleakage	Premolar teeth	Zirconomer	High viscosity GIC	15	Dye absorbance	Methylene blue	Spectrophotometry
Ranadheer E/2018 [30]	In vitro study	Microleakage	Premolar teeth	Zirconomer	Silver reinforced GIC	10	Dye penetration	Methylene blue	Stereomicroscopy
Mohammed Salman K/2019 [31]	In vitro study	Microleakage	Premolar teeth	Zirconomer-improved	Giomer	15	Dye penetration	Methylene blue	Stereomicroscopy
					RMGIC				
					Nano ionomer				
Mahmoud N/2020 [32]	In vitro study	Microleakage	Molar teeth	Zirconomer-improved	Glass hybrid	10	Dye penetration	Methylene blue	Stereomicroscopy
					RMGIC				
Sharafeddin F/2019 [33]	In vitro study	Microleakage	Molar teeth	Zirconomer	RMGIC	5	Dye penetration	Basic fuchsine	Stereomicroscopy
Meral E/2019 [34]	In vitro study	Microleakage	Molar teeth	Zirconomer	Glass hybrid	8	Dye penetration	Basic fuchsine	Stereomicroscopy
					Glass Carbomer				
Ashok L/2017–2020 [35]	In vitro study	Microleakage	Premolar teeth	Zirconomer-improved	Giomer	8	Dye penetration	Silver nitrate	Stereomicroscopy
Kaladevi M/2017–2020 [36]	In vitro study	Microleakage	Premolar teeth	Zirconomer-improved	High viscosity GIC	10	Dye Penetration	Silver paint	SEM
					Silver reinforced GIC				
Walia R/2016 [21]	Comparative cross-sectional study	Microleakage	Premolar teeth	Zirconomer	High viscosity GIC	15	Dye penetration	Methylene blue	Stereomicroscopy

GIC: glass ionomer cement; RMGIC: resin-modified glass ionomer cement; Amalgomer CR: ceramic-reinforced glass ionomer cement; SEM: scanning electron microscopy.

**Table 3:** Matrix of results from the NMA for compressive strength.

Comparison Compound										
	Amalgomer CR	−64.73 (−119.78; −9.68)	−65.85 (−103.93; −27.78)	10.76 (−43.66; 65.18)	−6.59 (−59.73; 46.55)	−12.87 (−67.60; 41.86)	−21.63 (−74.95; 31.69)		1.03 (−53.59; 55.65)	−3.44 (−56.90; 50.02)
	−119.76 (−161.84; −77.68)	Compomer *	75.58 (34.52; 116.64)		58.14 (2.86; 113.42)	161.90 (104.04; 219.76)	43.10 (−12.35; 98.55)		186.20 (130.41; 241.99)	61.29 (5.71; 116.87)
	−56.20 (−91.20; −21.19)	63.56 (26.58; 100.55)	Giomer *	117.54 (62.97; 172.11)	20.88 (−32.36; 74.12)	79.69 ( 47.48; 111.89)	5.84 (−47.57; 59.25)		66.16 (34.28; 98.03)	24.03 (−29.52; 77.58)
	32.24 (−17.09; 81.58)	152.00 (97.52; 206.48)	88.44 (41.84; 135.04)	Glass Carbomer		−23.63 (−78.20; 30.94)	.		−9.73 (−64.19; 44.73)	
Intervention compound	−26.26 (−68.86; 16.34)	93.49 (49.38; 137.61)	29.93 (−10.19; 70.05)	−58.51 (−115.14; −1.87)	Glass hybrid		4.31 (−33.17; 41.78)			−8.20 (−45.71; 29.31)
	24.60 (−11.41; 60.62)	144.36 (106.83; 181.89)	80.80 (53.13; 108.47)	−7.64 (−53.68; 38.40)	50.87 (9.15; 92.58)	High viscosity GIC		−46.90 (−99.78; 5.98)	−25.48 (−47.29; −3.67)	−40.70 (−93.11; 11.71)
	−21.89 (−64.58; 20.79)	97.86 (53.67; 142.06)	34.30 (−5.91; 74.51)	−54.14 (−110.83; 2.55)	4.37 (−33.10; 41.84)	−46.50 (−88.29; −4.71)	RMGIC			−12.65 (−50.23; 24.92)
	−6.69 (−63.81; 50.43)	113.07 (55.03; 171.11)	49.51 (−2.74; 101.76)	−38.93 (−102.86; 24.99)	19.57 (−41.29; 80.44)	−31.29 (−78.29; 15.71)	15.21 (−45.72; 76.13)	Silver reinforced GIC	−9.44 (−62.18; 43.30)	.
	−0.68 (−36.59; 35.22)	119.07 (81.79; 156.35)	55.51 (28.03; 82.99)	−32.93 (−78.90; 13.05)	25.58 (−16.02; 67.18)	−25.29 (−47.09; −3.48)	21.21 (−20.47; 62.89)	6.00 ( −40.97; 52.98)	Zirconomer	19.69 (−32.73; 72.11)
	−25.33 (−63.87; 13.21)	94.43 (54.31; 134.55)	30.87 (−3.72; 65.45)	−57.57 (−109.84; −5.31)	0.93 (−34.92; 36.79)	−49.93 (−83.98; −15.89)	−3.43 (−39.36; 32.49)	−18.64 (−74.55; 37.26)	−24.64 (−58.59; 9.30)	Zirconomer-improved

The numerical values within the cells represent the values of the intervention compound in relation to a specific comparison compound. Each row and column corresponds to the difference in compressive strength values, measured in megapascals (MPa). The values situated to the left or below indicate indirect estimates, while those positioned to the right or above signify direct estimates from the conducted studies. For example, the compressive strength value of Amalgomer CR is 119.76 MPa lower than that of compomer. Similarly, the compressive strength value of zirconomer is 24.64 MPa less than that of zirconomer-improved. In instances where no direct study exists comparing two specific compounds (denoted by values on the right or in cells above), these cells have been left empty. Notably, zirconia-reinforced glass ionomer cement exhibits significantly lower compressive strength than compomer and giomer. * GIC—glass ionomer cement; RMGIC—resin-modified glass ionomer cement; Amalgomer CR—ceramic-reinforced glass ionomer cement.

**Table 4:** Matrix of results from the NMA for microleakage.

Comparison Compound									
	Giomer			0.82 (−0.66; 2.30)	1.04 (−0.43; 2.50)	0.77 (−0.72; 2.25)		0.53 (−0.98; 2.04)	−0.06 (−1.12; 1.01)
	−0.89 (−3.06; 1.27)	Glass Carbomer	1.37 (−0.73; 3.47)					1.42 (−0.73; 3.57)	
	0.47 (−1.02; 1.96)	1.36 (−0.61; 3.33)	Glass hybrid			0.16 (−1.57; 1.90)		0.05 (−2.05; 2.15)	−0.40 (−2.13; 1.33)
	0.69 (−0.67; 2.05)	1.58 (−0.75; 3.92)	0.22 (−1.59; 2.03)	High viscosity GIC			10.22 (4.36; 16.07)	−0.66 ( −2.10; 0.79)	0.89 (−6.28; 8.06)
Intervention compound	0.93 (−0.41; 2.26)	1.82 (−0.58; 4.22)	0.46 (−1.31; 2.23)	0.24 (−1.59; 2.06)	Nano ionomer	−0.27 (−1.75; 1.21)			−0.80 (−2.27; 0.67)
	0.72 (−0.38; 1.81)	1.61 (−0.54; 3.76)	0.25 (−1.14; 1.63)	0.03 (−1.55; 1.61)	−0.21 (−1.57; 1.15)	RMGIC		−0.65 (−2.60; 1.30)	−0.54 (−1.65; 0.56)
	0.40 (−1.41; 2.22)	1.30 (−1.15; 3.74)	−0.06 (−2.08; 1.96)	−0.28 (−2.19; 1.62)	−0.52 (−2.65; 1.61)	−0.31 (−2.18; 1.55)	Silver reinforced GIC	0.90 (−0.58; 2.38)	−9.32 (−16.45; −2.20)
	0.54 (−0.63; 1.71)	1.43 (−0.56; 3.42)	0.07 (−1.38; 1.52)	−0.15 (−1.49; 1.18)	−0.39 (−2.01; 1.23)	−0.18 (−1.42; 1.06)	0.13 (−1.30; 1.56)	Zirconomer	
	−0.04 (−1.01; 0.93)	0.85 (−1.32; 3.02)	−0.51 (−1.91; 0.90)	−0.73 (−2.29; 0.83)	−0.97 (−2.29; 0.35)	−0.76 (−1.78; 0.26)	−0.45 (−2.34; 1.45)	−0.58 (−1.88; 0.72)	Zirconomer-improved

The numerical values within the cells represent the values of the intervention compound in relation to a specific comparison compound. Each row and column corresponds to the difference in mean microleakage values. The values situated to the left or below indicate indirect estimates, while those positioned to the right or above signify direct estimates from the conducted studies. For instance, the mean microleakage value of giomer is 0.89 times lower than that of glass carbomer. Where no direct study exists comparing two specific compounds (denoted by values on the right or in cells above), these cells have been left empty. GIC: glass ionomer cement; RMGIC: resin-modified glass ionomer cement; Amalgomer CR: ceramic-reinforced glass ionomer cement.

## Data Availability

Not applicable.

## Supplementary Materials

The following supporting information can be downloaded at: https://www.mdpi.com/article/10.3390/dj11090211/s1, Figure S1: Graphical plots of direct and indirect comparisons for: (A) Compressive Strength and (B) Microleakage; Figure S2: Comparisons of both direct and indirect measures: (A) Compressive Strength and (B) Microleakage; Figure S3: Heat map illustrating: (A) Compressive Strength and (B) Microleakage; Table S1: Search approach; Table S2: Generic name, brand name, and composition of the cement; Table S3: Incorporation of individual study data (intervention and comparison means) for Compressive Strength and Microleakage in the Network Meta-Analysis; Table S4: Ranking of materials based on: (A) Compressive Strength and (B) Microleakage; Table S5: Evaluation of Bias Risk and Individual Study Quality using JBI Critical Appraisal Tools, with Adjustments following the Checklist for Reporting In vitro Studies (CRIS Guidelines).
