# Supplementary material for: A Comprehensive Evaluation of Zirconia-Reinforced Glass Ionomer Cement’s Effectiveness in Dental Caries: A Systematic Review and Network Meta-Analysis
Source: Dent J (Basel). 2023 Sep 8;11(9):211. doi: 10.3390/dj11090211 (PMC10529821; doi:10.3390/dj11090211)
Supplement: Supplementary file 1 [file dentistry-11-00211-s001.zip › dentistry-2476625-supplementary.pdf]

Supplementary Materials

Figure S1. Graphical plots of direct and indirect comparisons for: A) Compressive Strength and B) Microleakage.

A) Compressive Strength

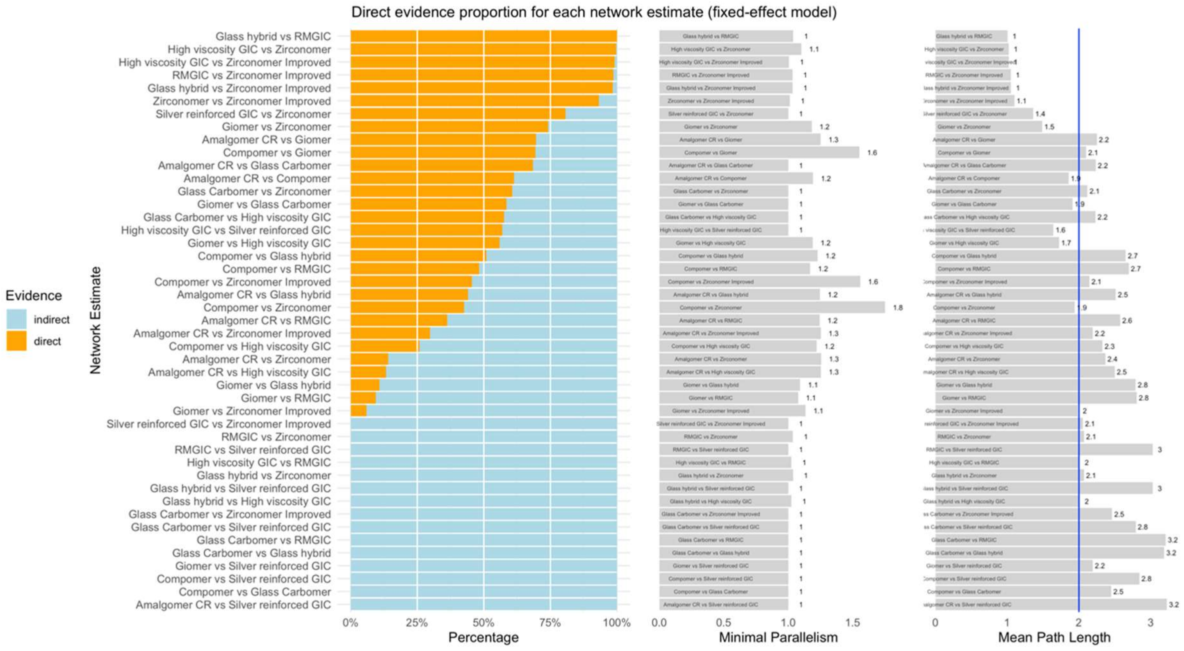

GIC: Glass Ionomer Cement; RMGIC: Resin Modified Glass Ionomer Cement; Amalgomer CR: Ceramic Reinforced Glass Ionomer Cement.

B) Microleakage

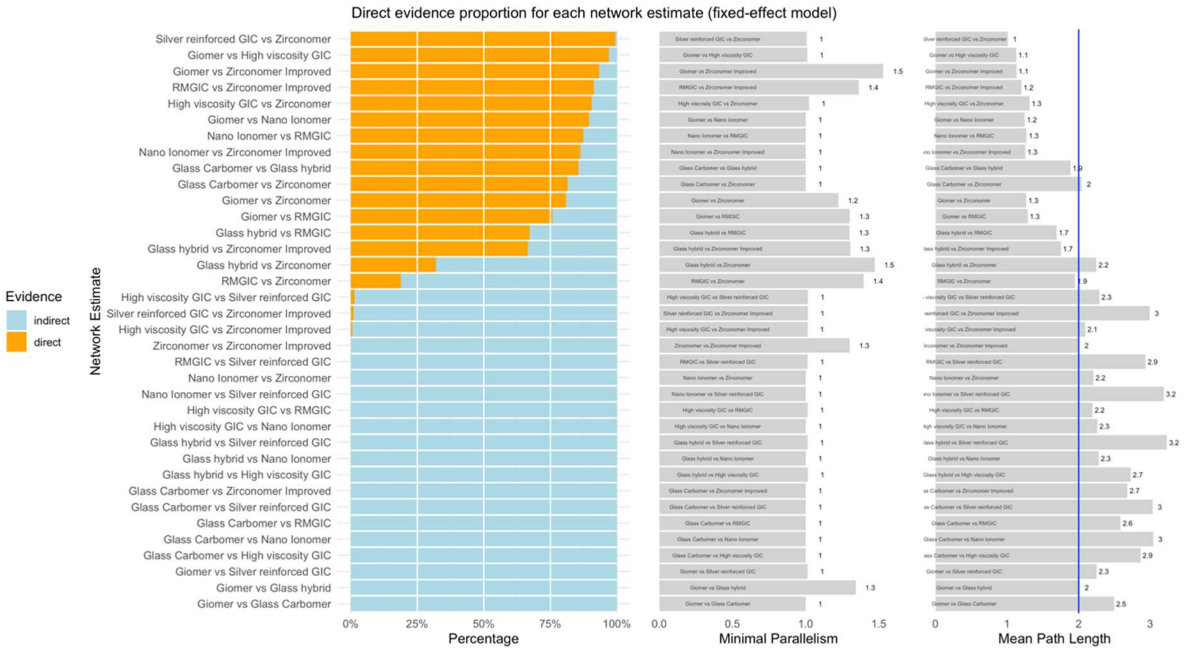

*GIC: Glass Ionomer Cement; RMGIC: Resin Modified Glass Ionomer Cement; Amalgomer CR: Ceramic Reinforced Glass Ionomer Cement.

Figure S2. Comparisons of both direct and indirect measures: A) Compressive Strength and B) Microleakage.

A) Compressive Strength

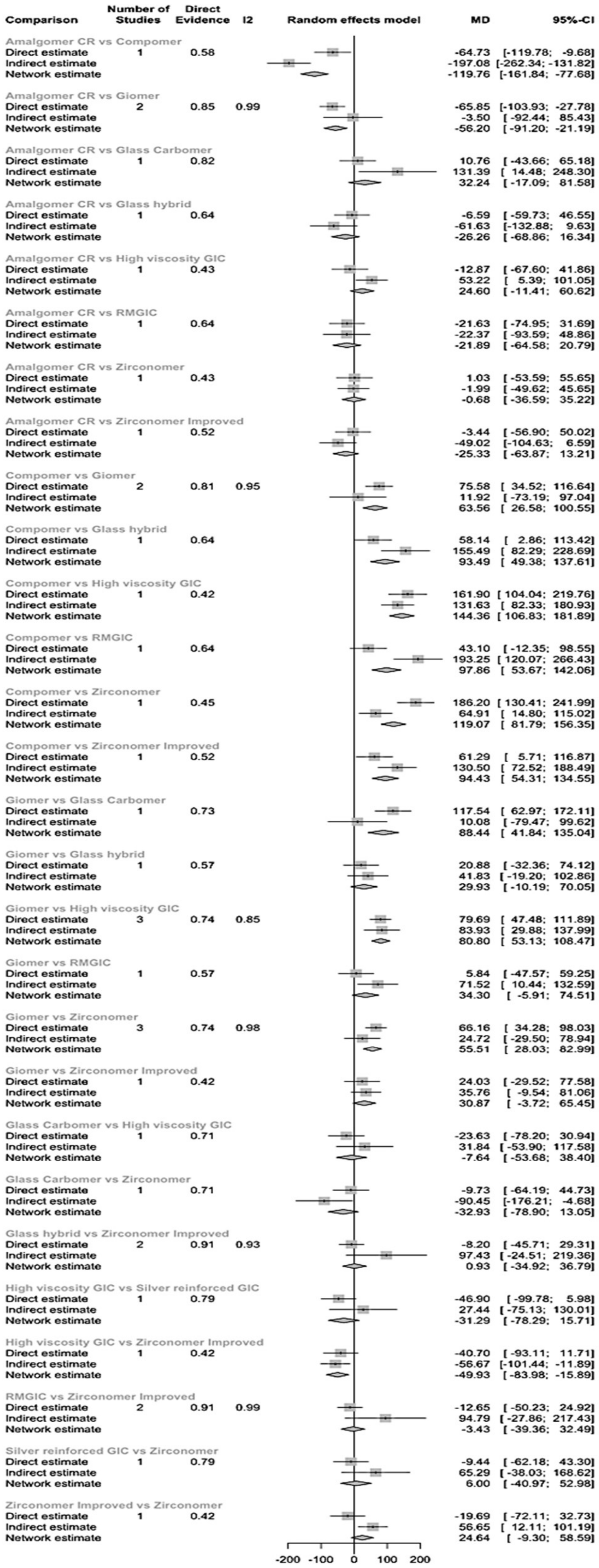

* GIC: Glass Ionomer Cement; RMGIC: Resin Modified Glass Ionomer Cement;
Amalgomer CR: Ceramic Reinforced Glass Ionomer Cement.

B) Microleakage

Comparison	Number of Studies	Direct Evidence	I2	Random effects model	MD	95%-CI
Giomer vs High viscosity GIC						
Direct estimate	1	0.85			0.82	[-0.66; 2.30]
Indirect estimate					-0.03	[-3.49; 3.43]
Network estimate					0.69	[-0.67; 2.05]
Giomer vs Nano Ionomer						
Direct estimate	1	0.83			1.04	[-0.43; 2.50]
Indirect estimate					0.39	[-2.87; 3.64]
Network estimate					0.93	[-0.41; 2.26]
Giomer vs RMGIC						
Direct estimate	1	0.54			0.77	[-0.72; 2.25]
Indirect estimate					0.66	[-0.96; 2.28]
Network estimate					0.72	[-0.38; 1.81]
Giomer vs Zirconomer						
Direct estimate	1	0.60			0.53	[-0.98; 2.04]
Indirect estimate					0.54	[-1.30; 2.39]
Network estimate					0.54	[-0.63; 1.71]
Giomer vs Zirconomer Improved						
Direct estimate	2	0.83	0.63		-0.06	[-1.12; 1.01]
Indirect estimate					0.03	[-2.33; 2.39]
Network estimate					-0.04	[-1.01; 0.93]
Glass Carbomer vs Glass hybrid						
Direct estimate	1	0.88			1.37	[-0.73; 3.47]
Indirect estimate					1.30	[-4.39; 6.98]
Network estimate					1.36	[-0.61; 3.33]
Glass Carbomer vs Zirconomer						
Direct estimate	1	0.86			1.42	[-0.73; 3.57]
Indirect estimate					1.49	[-3.89; 6.87]
Network estimate					1.43	[-0.56; 3.42]
Glass hybrid vs RMGIC						
Direct estimate	1	0.64			0.16	[-1.57; 1.90]
Indirect estimate					0.41	[-1.90; 2.71]
Network estimate					0.25	[-1.14; 1.63]
Glass hybrid vs Zirconomer						
Direct estimate	1	0.48			0.05	[-2.05; 2.15]
Indirect estimate					0.09	[-1.92; 2.09]
Network estimate					0.07	[-1.38; 1.52]
Glass hybrid vs Zirconomer Improved						
Direct estimate	1	0.66			-0.40	[-2.13; 1.33]
Indirect estimate					-0.72	[-3.11; 1.68]
Network estimate					-0.51	[-1.91; 0.90]
High viscosity GIC vs Silver reinforced GIC						
Direct estimate	1	0.11			10.22	[4.36; 16.07]
Indirect estimate					-1.53	[-3.55; 0.49]
Network estimate					-0.28	[-2.19; 1.62]
High viscosity GIC vs Zirconomer						
Direct estimate	2	0.86	0.64		-0.66	[-2.10; 0.79]
Indirect estimate					2.88	[-0.66; 6.43]
Network estimate					-0.15	[-1.49; 1.18]
High viscosity GIC vs Zirconomer Improved						
Direct estimate	1	0.05			0.89	[-6.28; 8.06]
Indirect estimate					-0.81	[-2.41; 0.79]
Network estimate					-0.73	[-2.29; 0.83]
Nano Ionomer vs RMGIC						
Direct estimate	1	0.84			-0.27	[-1.75; 1.21]
Indirect estimate					0.10	[-3.25; 3.45]
Network estimate					-0.21	[-1.57; 1.15]
Nano Ionomer vs Zirconomer Improved						
Direct estimate	1	0.81			-0.80	[-2.27; 0.67]
Indirect estimate					-1.67	[-4.66; 1.33]
Network estimate					-0.97	[-2.29; 0.35]
RMGIC vs Zirconomer						
Direct estimate	1	0.40			-0.65	[-2.60; 1.30]
Indirect estimate					0.14	[-1.47; 1.74]
Network estimate					-0.18	[-1.42; 1.06]
RMGIC vs Zirconomer Improved						
Direct estimate	2	0.85	0.00		-0.54	[-1.65; 0.56]
Indirect estimate					-2.00	[-4.67; 0.66]
Network estimate					-0.76	[-1.78; 0.26]
Silver reinforced GIC vs Zirconomer						
Direct estimate	1	0.93			0.90	[-0.58; 2.38]
Indirect estimate					-10.26	[-15.70; -4.82]
Network estimate					0.13	[-1.30; 1.56]
Silver reinforced GIC vs Zirconomer Improved						
Direct estimate	1	0.07			-9.32	[-16.45; -2.20]
Indirect estimate					0.23	[-1.74; 2.20]
Network estimate					-0.45	[-2.34; 1.45]

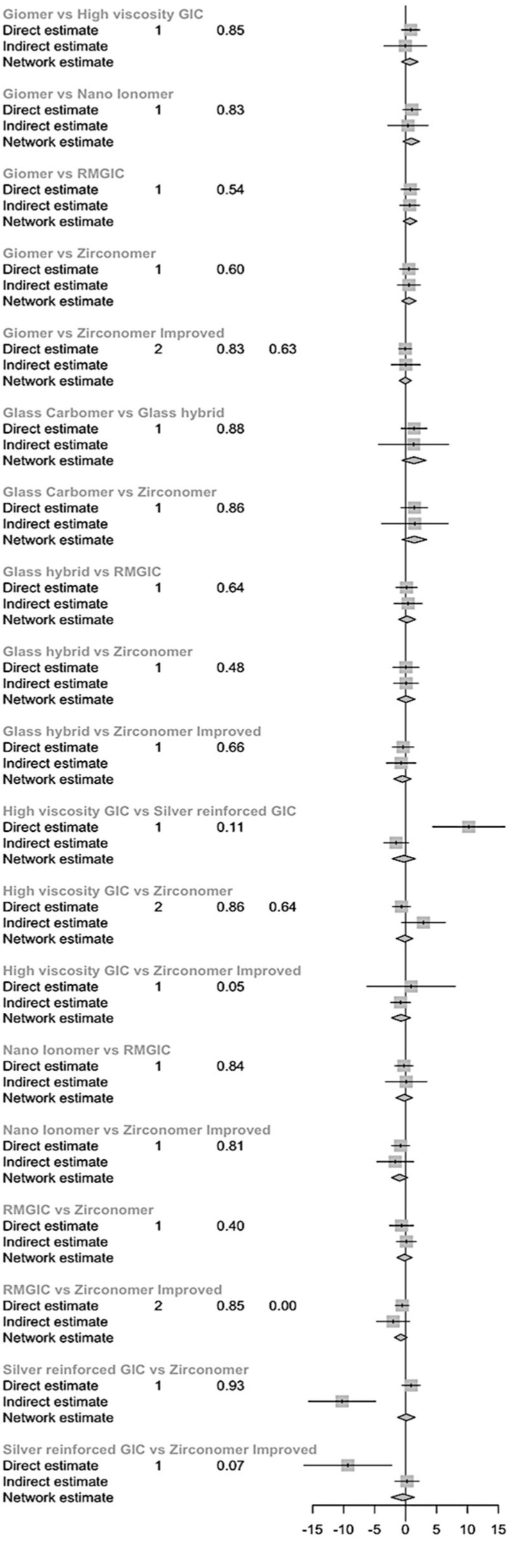

*GIC: Glass Ionomer Cement; RMGIC: Resin Modified Glass Ionomer Cement; Amalgomer CR: Ceramic Reinforced Glass Ionomer Cement.

Figure S3. Heat map illustrating: A) Compressive Strength and B) Microleakage.

A) Compressive Strength

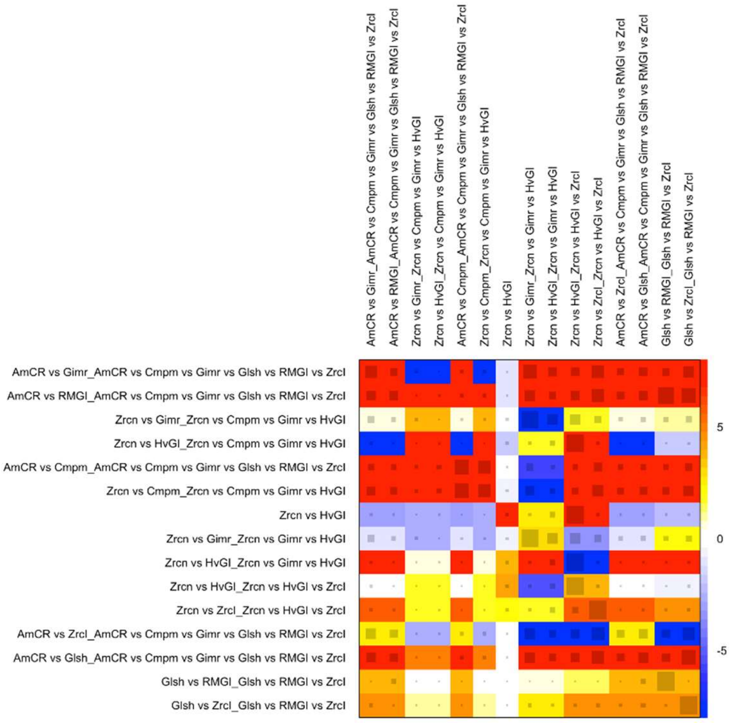

B) Microleakage

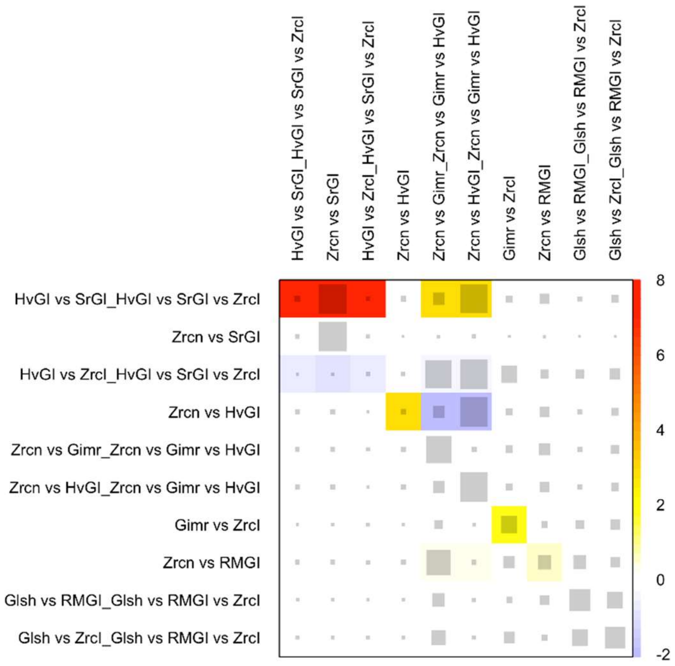

Table S1. Search approach.

**PubMed-MEDLINE**
('cement specimen' OR 'tooth'/exp OR 'dental evolution' OR 'dentes' OR 'teeth' OR 'tooth' OR 'tooth auxiliary' OR 'tooth components' OR 'tooth condition' OR 'tooth emergency' OR teeth OR tooth) AND ('zirconomer'/exp OR zirconia OR 'zirconium'/exp OR 'novel glass ionomer cement' OR 'zirconia reinforced glass ionomer cement') AND ('tooth cement'/exp OR 'calcium hydroxide cement' OR 'calcium hydroxide dental cement' OR 'calcium hydroxide tooth cement' OR 'cement, dental' OR 'dental cement' OR 'dental cements' OR 'ethoxy benzoic acid dental cement' OR 'fatty acid dental cement' OR 'tooth cement' OR 'cement'/exp OR 'adhesor cement' OR 'canal cement' OR 'cement' OR 'cement granule' OR 'cementing substance' OR 'cermet cements' OR 'coe tray cement' OR 'portland 500' OR 'miracle mix'/exp OR 'modified glass ionomer cements' OR 'fuji ix'/exp OR 'high viscosity glass ionomer cement' OR 'giomer'/exp OR compomers OR 'reinforced glass ionomer cement' OR 'nano ionomer' OR 'glass ionomer cements') AND ('*in vitro* study'/exp OR '*in vitro*' OR '*in vitro* studies' OR '*in vitro* study' OR '*in vitro* technique' OR '*in vitro* techniques')

**Embase**
('cement specimen' OR 'tooth'/exp OR 'dental evolution' OR 'dentes' OR 'teeth' OR 'tooth' OR 'tooth auxiliary' OR 'tooth components' OR 'tooth condition' OR 'tooth emergency' OR teeth OR tooth) AND ('zirconomer'/exp OR zirconia OR 'zirconium'/exp OR 'novel glass ionomer cement' OR 'zirconia reinforced glass ionomer cement') AND ('tooth cement'/exp OR 'calcium hydroxide cement' OR 'calcium hydroxide dental cement' OR 'calcium hydroxide tooth cement' OR 'cement, dental' OR 'dental cement' OR 'dental cements' OR 'ethoxy benzoic acid dental cement' OR 'fatty acid dental cement' OR 'tooth cement' OR 'cement'/exp OR 'adhesor cement' OR 'canal cement' OR 'cement' OR 'cement granule' OR 'cementing substance' OR 'cermet cements' OR 'coe tray cement' OR 'portland 500' OR 'miracle mix'/exp OR 'modified glass ionomer cements' OR 'fuji ix'/exp OR 'high viscosity glass ionomer cement' OR 'giomer'/exp OR compomers OR 'reinforced glass ionomer cement' OR 'nano ionomer' OR 'glass ionomer cements') AND ('*in vitro* study'/exp OR '*in vitro*' OR '*in vitro* studies' OR '*in vitro* study' OR '*in vitro* technique' OR '*in vitro* techniques')

Table S2. Generic name, brand name, and composition of the cement.

Srl no.	Cement (Generic name referenced in this review)	Brand (As mentioned in the original article)	Composition
1	Zirconia reinforced glass ionomer cement	Zirconomer (Shofu, Japan)	Powder: zirconium oxide, glass powder, tartaric acid (1 - 10%), polyacrylic acid (20 - 50%). Liquid: deionized water.
2	Zirconia reinforced glass ionomer cement	Zirconomer improved (Shofu, Japan)	Powder: zirconium oxide (zirconia nano fillers), glass powder, tartaric acid (1 - 10%), polyacrylic acid (20 - 50%). Liquid: deionized water.
		Ketac molar (3M ESPE, USA)	Powder: calcium aluminium lanthanum fluorosilicate glass, copolymer, pigments. Liquid: acrylic acid, maleic acid copolymer, tartaric acid, benzoic acid.
3	High viscosity GIC	Type IX, Type IX extra (GC, Tokyo, Japan)	Powder: aluminosilicate glass, polyacrilic acid. Liquid: polyacrilic acid, polybasic acid.
4	Giomer	Beautifil II (Shofu, Kyoto, Japan)	Bis-GMA, TEGDMA, aluminium oxide, silica, aluminofluoro-borosilicate glass filler, pre-reacted glass ionomer filler, camphoroquinone.
		Miracle mix (GC, Tokyo, Japan)	powder: aluminosilicate glass, silver alloy powder. Liquid: polyacrylic acid, water, polybasic carboxylic acid.
5	Silver reinforced GIC	Xtracem-S (Medicept Dental)	Powder: fluoro alumina silicate glass, nano silver filler. Liquid: polyacrylic acid, tartaric acid.
6	RMGIC	Fuji II LC (GC, Tokyo, Japan)	Powder: aluminosilicate glass, pigments. Liquid: polyacrylic acids, distilled water, HEMA(17%), dimethacrylate monomer, camporoquinone.

		Dyract-XP (Dentsply sirona, Germany)	Sr-Al-N-Fluouroaluminosilicate glass, strontium fluoride, UDMA, TEGDMA, TMPTMA, TCB RESIN, dimethacrylate resins, dimethylaminobenzoic acid ethyl ester, camphorquinone, BHT, UV stabilizer, iron oxide and titanium oxide pigments.
7	Compomer	Compoglass F (Ivoclar vivadent, NY)	Barium fluorosilicate glass, UDMA, TEGDMA, CADCADM, ytterbium trifluoride.
8	Glass Hybrid	Equia forte (GC, Tokyo, Japan)	Powder: strontium-fluoro-alumino-silicate glass, polyacrylic acid.<br>Liquid: aqueous polyacrylic acid.
9	Glass carbomer	Glassfill (GCP, Netherland)	Powder: Nanofluro, hydroxyapatite.<br>Liquid: polyacids, silica.
10	Amalgomer CR	Amalgomer CR (Advanced Healthcare Ltd., UK)	Powder: fluoro-alumino-silicate glass, polyacrylic acid powder, tartaric acid powder, ceramic reinforcing powder.<br>Liquid: polyacrylic acid, distilled water.
11	Nano-Ionomer	Ketac N 100 (3M ESPE, St. Paul, MN, USA)	Deionized water, HEMA, VBCP, fluoroaluminosilicate glass, nanomers, nanoclusters.

GIC: Glass ionomer cement; **RMGIC**: Resin Modified Glass Ionomer Cement; **UDMA**: Urethane dimethacrylate; **HEMA**: Hydroxyethyl methacrylate; **TEGDMA**: Triethylene glycol dimethacrylate; **Bis-GMA**: Bis-phenol A glycidyl methacrylate; **CADCADM**: Cycloaliphatic dicarboxylic acid dimethacrylate; **VBCP**: Vitrebond Copolymer.

Table S3. Incorporation of individual study data (intervention and comparison means) for Compressive Strength and Microleakage in the Network Meta-Analysis.

Sr. No.	Author	Year	Intervention	Comparative	Mean_Intervention	Mean_Comparison
			**Compressive Strength**			
1	Walia R [21]	2016	Zirconomer	High viscosity GIC	220.48	153.74
1	Walia R [21]	2016	Zirconomer	Giomer	220.48	250.95
2	Bhatia H [22]	2017	Zirconomer	High viscosity GIC	160.91	104.57
2	Bhatia H [22]	2017	Zirconomer	Silver reinforced GIC	160.91	151.47
3	Canturk K [23]	2020	Zirconomer Improved	RMGIC	66.7	84.89
3	Canturk K [23]	2020	Zirconomer Improved	Compomer	66.7	127.99
3	Canturk K [23]	2020	Zirconomer Improved	Glass Hybrid	66.7	69.85
3	Canturk K [23]	2020	Zirconomer Improved	Giomer	66.7	90.73
3	Canturk K [23]	2020	Zirconomer Improved	Amalgomer CR	66.7	63.26
4	Shetty C [24]	2017	Zirconomer	High viscosity GIC	321.92	261.53
4	Shetty C [24]	2017	Zirconomer Improved	High viscosity GIC	302.23	261.53
5	Patel A [25]	2018	Zirconomer	High viscosity GIC	18.05	18.295
6	Patil K [26]	2016	Zirconomer	Giomer	12.6	75.5
6	Patil K [26]	2016	Zirconomer	High viscosity GIC	12.6	36.9
6	Patil K [26]	2016	Zirconomer	Compomer	12.6	198.8
7	S Dhivya [27]	2017-2020	Zirconomer Improved	Glass Hybrid	153.777	134.756
7	S Dhivya [27]	2017-2020	Zirconomer Improved	RMGIC	153.777	111.892
8	Uğurlu M [28]	2020	Zirconomer	Glass Carbomer	140.55	130.82
8	Uğurlu M [28]	2021	Zirconomer	Amalgomer CR	140.55	141.58

8	Uğurlu M [28]	2022	Zirconomer	High viscosity GIC	140.55	154.45
8	Uğurlu M [28]	2023	Zirconomer	Giomer	140.55	248.36
			**Microleakage**			
1	Asafarlal S [29]	2017	Zirconomer	High viscosity GIC	9.88	5.7622
2	Ranadheer E [30]	2018	Zirconomer	Silver reinforced GIC	1.40	2.30
3	Mohammed Salman K [31]	2019	Zirconomer Improved	Giomer	2.30	2.53
3	Mohammed Salman K [31]	2019	Zirconomer Improved	RMGIC	2.30	1.77
3	Mohammed Salman K [31]	2019	Zirconomer Improved	Nano Ionomer	2.30	1.50
4	Mahmoud N [32]	2020	Zirconomer Improved	Glass Hybrid	2.21	1.81
4	Mahmoud N [32]	2020	Zirconomer Improved	RMGIC	2.21	1.65
5	Sharafeddin F [33]	2019	Zirconomer	RMGIC	1.85	1.20
6	Meral E [34]	2019	Zirconomer	Glass Hybrid	1.58	1.63
6	Meral E [34]	2019	Zirconomer	Glass Carbomer	1.58	3.00
7	Ashok L [35]	2017-2020	Zirconomer Improved	Giomer	1.44	1.07
8	Kaladevi M [36]	2017-2020	Zirconomer Improved	High viscosity GIC	15.80	16.69
8	Kaladevi M [36]	2017-2020	Zirconomer Improved	Silver reinforced GIC	15.80	6.48
9	Walia R [21]	2016	Zirconomer	High viscosity GIC	1.67	1.38
9	Walia R [21]	2016	Zirconomer	Giomer	1.67	2.20

*GIC: Glass Ionomer Cement; RMGIC: Resin Modified Glass Ionomer Cement; Amalgomer CR: Ceramic Reinforced Glass Ionomer Cement.

Table S4. Ranking of materials based on: A) Compressive Strength and B) Microleakage.

A) Compressive strength

Cement	P Score
Compomer	0.9999
Giomer	0.8676
Glass hybrid	0.6290
Zirconomer improved	0.6271
RMGIC	0.5787
Silver reinforced GIC	0.4269
Zirconomer	0.3528
Amalgomer cr	0.3416
High viscosity GIC	0.0945
Glass carbomer	0.0819

*GIC: Glass Ionomer Cement; RMGIC: Resin Modified Glass Ionomer Cement;
Amalgomer CR: Ceramic Reinforced Glass Ionomer Cement.

B) Microleakage

Cement	P score
Nano ionomer	0.7562
RMGIC	0.6913
High viscosity GIC	0.6566
Zirconomer	0.5961
Glass hybrid	0.5579
Silver reinforced GIC	0.5218
Giomer	0.3073
Zirconomer improved	0.2907
Glass carbomer	0.1221

*GIC: Glass Ionomer Cement; RMGIC: Resin Modified Glass Ionomer Cement;
Amalgomer CR: Ceramic Reinforced Glass Ionomer Cement.

[NOTE: The P score arranges the materials from highest to lowest based on their effect.]

Table S5. Evaluation of Bias Risk and Individual Study Quality using Joanna Briggs Institute (JBI) Critical Appraisal Tools, with Adjustments following the Checklist for Reporting *In vitro* Studies (CRIS Guidelines).

STUDY	Q1. Was the sample size calc - ulation Adeq -uately Repo -rted?	Q2. Was true random-isation used for assi -gnment of samples to treat -ment groups?	Q3. Were treat-ment groups similar at baseline?	Q4. Were Trea -tment groups treated by the same operator?	Q5. Were those deliv -ering treat -ment blind to treat -ment Assign -ment?	Q6. Were Treat -ment groups treated iden -tically other than the inter -enetion of interest?	Q7. Were outcomes assessed by the same out -come assessor or were the out -come assessors calibrated and the level of agree -ment Deter -mined?	Q8. Were Out -come Asse -ssors blind to treat -ment Assign -ment?	Q9. Were Out -comes measured in the same manner for treat -ment groups?	Q10. Was appro -priate stati -stical analysis used?	Q11. Were results for all samples repo -rted? If not, were the losses adeq -uately desc -ribed and ana -lysed?	Q12. Was the study design appr -opriate and appa -rently free of other biases?	Yes (n)	Risk of Bias
Walia R 2016 [21]	no	unclear	yes	unclear	no	yes	Unclear	no	yes	yes	yes	yes	6	MODERATE
Bhatia H 2017 [22]	no	unclear	yes	unclear	no	yes	unclear	no	yes	yes	yes	yes	6	MODERATE
Canturk K 2020 [23]	no	unclear	yes	unclear	no	yes	unclear	no	yes	yes	yes	yes	6	MODERATE
Shetty C 2017 [24]	no	unclear	yes	unclear	no	yes	unclear	no	yes	yes	yes	no	5	HIGH
Patel A 2018 [25]	no	unclear	yes	unclear	no	yes	unclear	no	yes	yes	yes	yes	6	MODERATE
Patil K 2016 [26]	no	unclear	yes	unclear	no	yes	unclear	no	yes	yes	yes	yes	6	MODERATE
S Dhivya 2017-2020 [27]	no	unclear	yes	unclear	no	yes	unclear	no	yes	yes	yes	yes	6	MODERATE
Uğurlu M 2020 [28]	no	unclear	yes	unclear	no	yes	unclear	no	yes	yes	yes	yes	6	MODERATE
Asafarlal S 2017 [29]	no	unclear	yes	yes	no	yes	unclear	yes	yes	yes	yes	yes	8	LOW
Ranadheer E 2018 [30]	no	unclear	yes	yes	no	yes	unclear	no	yes	yes	yes	yes	7	MODERATE
Mohammed Salman K 2019 [31]	no	unclear	yes	unclear	no	yes	unclear	no	yes	yes	yes	no	5	HIGH
Mahmoud N 2020 [32]	no	unclear	yes	unclear	no	yes	unclear	yes	yes	yes	yes	yes	7	MODERATE
Sharafeddin F 2019 [33]	no	unclear	yes	unclear	no	yes	unclear	no	yes	yes	yes	yes	6	MODERATE
Meral E 2019 [34]	no	unclear	yes	yes	no	yes	unclear	no	yes	yes	yes	yes	7	MODERATE
Ashok L 2017-2020 [35]	no	unclear	yes	unclear	no	yes	unclear	no	yes	yes	yes	yes	6	MODERATE
Kaladevi M 2017-2020 [36]	no	unclear	yes	unclear	no	yes	unclear	no	yes	yes	yes	yes	6	MODERATE

* High risk of bias was assigned when the study achieved up to five 'yes' scores, moderate risk when the study achieved six to eight 'yes' scores, and low risk when the study achieved eight or more 'yes' scores.
